# Alterations of the Sialylation Machinery in Brugada Syndrome

**DOI:** 10.3390/ijms232113154

**Published:** 2022-10-29

**Authors:** Andrea Ghiroldi, Giuseppe Ciconte, Pasquale Creo, Adriana Tarantino, Dario Melgari, Sara D’Imperio, Marco Piccoli, Federica Cirillo, Emanuele Micaglio, Michelle M. Monasky, Anthony Frosio, Emanuela T. Locati, Gabriele Vicedomini, Ilaria Rivolta, Carlo Pappone, Luigi Anastasia

**Affiliations:** 1 IMTC—Institute of Molecular and Translational Cardiology, San Donato Milanese, 20097 Milan, Italy; 2 Laboratory of Stem Cells for Tissue Engineering, IRCCS Policlinico San Donato, San Donato Milanese, 20097 Milan, Italy; 3 Arrhythmology Department, IRCCS Policlinico San Donato, San Donato Milanese, 20097 Milan, Italy; 4 Faculty of Medicine and Surgery, University Vita-Salute San Raffaele, Via Olgettina 58, 20097 Milan, Italy; 5 School of Medicine and Surgery, Università degli Studi Milano-Bicocca, 20126 Milan, Italy

**Keywords:** Brugada Syndrome, sudden cardiac death, arrhythmias, ventricular tachycardia, sialylation, glycosylation, peripheral cells, PBMCs

## Abstract

Brugada Syndrome (BrS) is an inherited arrhythmogenic disorder with an increased risk of sudden cardiac death. Recent evidence suggests that BrS should be considered as an oligogenic or polygenic condition. Mutations in genes associated with BrS are found in about one-third of patients and they mainly disrupt the cardiac sodium channel NaV1.5, which is considered the main cause of the disease. However, voltage-gated channel’s activity could be impacted by post-translational modifications such as sialylation, but their role in BrS remains unknown. Thus, we analyzed high risk BrS patients (*n* = 42) and healthy controls (*n* = 42) to assess an involvement of sialylation in BrS. Significant alterations in gene expression and protein sialylation were detected in Peripheral Blood Mononuclear Cells (PBMCs) from BrS patients. These changes were significantly associated with the phenotypic expression of the disease, as the size of the arrhythmogenic substrate and the duration of epicardial electrical abnormalities. Moreover, protein desialylation caused a reduction in the sodium current in an in vitro NaV1.5-overexpressing model. Dysregulation of the sialylation machinery provides definitive evidence that BrS affects extracardiac tissues, suggesting an underlying cause of the disease. Moreover, detection of these changes at the systemic level and their correlation with the clinical phenotype hint at the existence of a biomarker signature for BrS.

## 1. Introduction

Brugada Syndrome is an inherited arrhythmogenic disorder associated with an increased risk of sudden cardiac death (SCD) [1,2,3,4]. The worldwide prevalence of 5–20 cases per 10,000 people in the population is probably underestimated because the diagnosis depends on a specific ECG pattern that is often difficult to recognize even for experienced clinicians [5]. BrS is a genetically heterogeneous channelopathy with autosomal dominant inheritance and incomplete penetrance [6]. However, causative mutations are only identified in 35% of cases [7], primarily disrupting the *SCN5A* gene, which encodes the alpha subunit of the cardiac sodium channel NaV1.5 [8]. The channel is activated during negative membrane voltage and is responsible for the rapid increase in cardiac action potential (AP) [9]. As a result, it plays a key role in the pathophysiology and severity of the disease [10]. There are more than 500 mutations of *SCN5A* described in BrS, most of which cause NaV1.5 loss of function [11]. However, intrinsic mutations in the *SCN5A* gene are found in only 20–25% of BrS patients [12], underscoring the current limited knowledge of the genetic basis and pathophysiological mechanism of the disease. Recently, it has been suggested that the occurrence of BrS requires structural abnormalities of the right ventricular outflow tract (RVOT) due to interstitial fibrosis and reduced conduction reserve due to the decrease in inward sodium current [13,14]. Thus, it is conceivable that there are other mechanisms besides *SCN5A* mutations that impair NaV1.5 activity and thus contribute to the reduction in conduction reserve. Indeed, multiple variables, including protein-protein interactions and post-translational modifications (PTMs), have been shown to affect sodium channel activity [9] and ultimately lead to cardiac dysfunction [15]. Several studies have found that NaV1.5 forms a macromolecular protein complex that is a target of PTMs whose alterations can cause pathological arrhythmias [15]. Because of its complexity, diversity, and control over a wide range of physiological and pathological functions, glycosylation is a key class of PTMs [16]. In particular, sialylation refers to the terminal addition of negatively charged sialic acids to oligosaccharides and glycoproteins [17]. Sialic acid glycosylates up to 30% of the mass of voltage-gated sodium channels [18], and changes in sodium channel sialylation have been found to affect the excitation state of cardiac and neural cells [19]. The biosynthetic pathways of sialic acids and sialylated glycans are ubiquitous and regulated by about 20 enzymes, with the main sialyltransferases localized in the Golgi [20]. A reduction in sialyltransferase activity has been found in heart failure, in which defective glycosylation of the sodium channel can lead to channel-dependent arrhythmogenesis [21]. Alterations in sialyltransferases were found in congenital disorders of glycosylation (CDGs), in which patients often develop cardiac dysfunction (i.e., dilated or hypertrophic cardiomyopathies with associated arrhythmias) [22]. Analysis of sialic acid bound to transferrin is a preliminary diagnostic test for CDGs [23]. Surprisingly, the involvement of sialylation in BrS remains unknown. Although alterations in plasma proteins have been reported in BrS patients [24,25], changes in the protein sialylation machinery or other PTMs have not been investigated.

## 2. Results

### 2.1. Study Population

A total of 84 patients (62 males, 73.8%; mean age 40.2 ± 15.5 years) were included in this study. The BrS group included 42 patients, whereas 42 healthy subjects served as the CTR group because of a negative ajmaline test. The Shanghai Score was calculated for each subject enrolled. All the BrS patients showed a score > 3.5 and 23 (54.8%) presented a score > 7. All the CTR subjects had a score > 2, and 6 (14.3%) had score > 3.5, but all of them resulted negative to the ajmaline challenge test. Among the BrS patients, 9 (10.7%) survived a previous cardiac arrest, and 32 (76.2%) had documented appropriate ICD therapies due to VAs in the BrS group. Nineteen subjects (45.2%) presented with a spontaneous type 1 pattern. Family history of sudden death was observed in 23 patients (54.7%) in the BrS group, and 22 (52.3%) were inducible for VT/VF at the EPS. Overall clinical characteristics are summarized in Table 1, comparing BrS and CTR groups.

### 2.2. Gene Expression Analysis Shows Alteration of the Sialylation Machinery in BrS Patients

**Figure 1:**
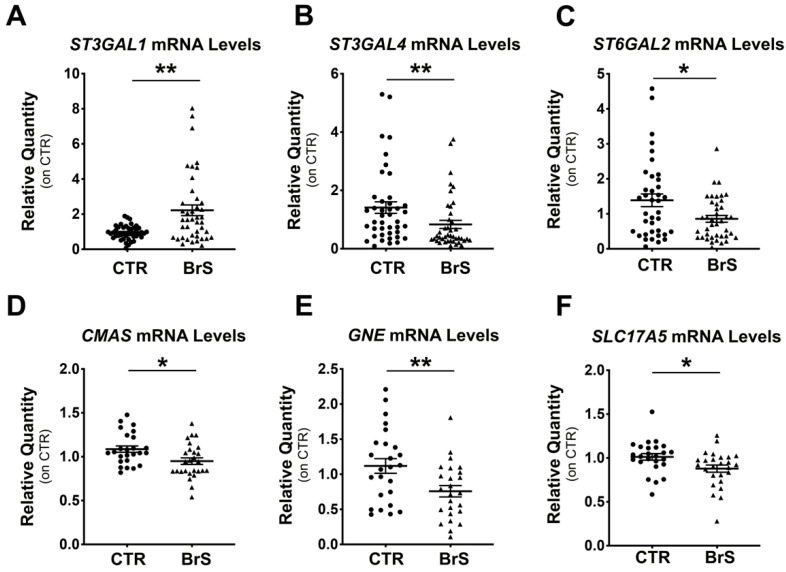
Gene expression analysis reveals dysregulations of sialylation pathway in BrS patients. PBMCs were isolated from healthy subjects (CTR) and BrS patients (BrS) and the expression of six genes involved in the sialylation pathway was evaluated. mRNA expression of *ST3Gal1* (**A**), *ST3Gal4* (**B**), *ST6Gal2* (**C**), *CMAS* (**D**), *GNE* (**E**), and *SLC17A5* (**F**) through RT-PCR. CTR: N = 42, BrS: N = 42 for (**A**–**C**). CTR: N = 25, BrS: N = 27 for (**D**–**F**). Data represent the mean ± SEM and are expressed as relative amounts compared with healthy controls. Each dot and triangles in the graphs represent a single healthy control and a single BrS patients, respectively. Statistical significance was determined by Student’s *t*-test or Mann–Whitney, based on the normal distribution of data. * *p* < 0.05, ** *p* < 0.01.

Real-time PCR analysis of *ST3Gal1*, *ST3Gal4*, *ST6Gal2*, *ST8Sia2*, and *ST8Sia5* was performed on mRNAs extracted from PBMCS of BrS patients (BrS) and compared with those from healthy controls (CTR). *ST3Gal1* showed a 2-fold increase, respectively, in BrS compared with CTR (Figure 1A), whereas *ST3Gal1* and *ST6Gal2* showed a 2- and 1.7-fold decrease, respectively (Figure 1B,C). *ST8Sia4* and *ST8Sia5* mRNAs could not be detected in PBMCs (data not shown). Real-Time PCR analysis of *CMAS*, *GNE*, and *SLC17A*, key genes of the sialic acid biosynthetic pathway, showed 1.2-, 2-, and 1.3-fold downregulation, respectively, in BrS patients compared with CTR (Figure 1D–F).

### 2.3. Flow Cytometric Analysis of PBMCs Reveals a Reduction in Membrane Protein Sialylation Levels

**Figure 2:**
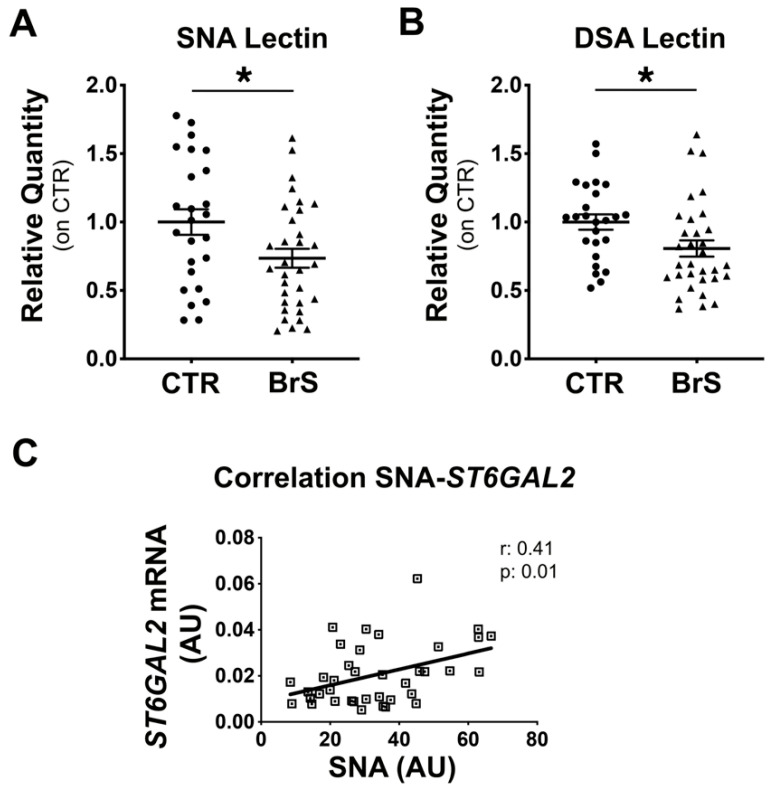
Sialylation analysis of membrane PBMCs proteins showed alterations in BrS patients. PBMCs isolated from healthy subjects (CTR) and BrS patients (BrS) were incubated with two specific lectins, SNA or DSA, to assess the sialylation or desialylation status of proteins, respectively and analyzed through flow cytometry. (**A**) Sialylated protein levels detected with the incubation with SNA lectin. (**B**) Desialylated protein levels detected with the incubation with DSA lectin. CTR: N = 25, BrS: N = 32. Data represent the mean ± SEM and are expressed as relative amounts compared with healthy controls. Statistical significance was determined by Student’s *t*-test. Each dot and triangles in the graphs represent a single healthy control and a single BrS patients, respectively. (**C**) The correlation between sialylation and *ST6Gal2* was analyzed by comparing RNA expression levels of ST6Gal2 and the SNA levels in PBMCs. Each square in the graphs represents a single subject. N = 38. * *p* < 0.05.

To assess the overall sialylation status of PBMC membrane proteins, flow cytometric analysis was performed using SNA and DSA lectins that specifically detect sialylated and desialylated proteins, respectively, as described in the Methods section. Results showed a 25% decrease in protein sialylation (Figure 2A) and a 15% decrease in protein desialylation (Figure 2B) in PBMCs from BrS compared with CTR. A positive association was observed between *ST6Gal2* gene expression and sialylated protein levels (Figure 2C), with a Pearson coefficient of 0.41 and a *p*-value of 0.01.

### 2.4. Western Blot Analysis of PBMCs Show a Decrease of Intracellular Protein Sialylation Levels in BrS Patients

**Figure 3:**
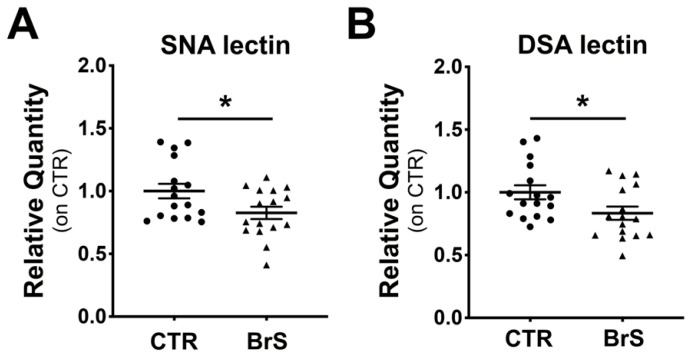
PBMCs intracellular proteins are differentially sialylated in BrS patients. Sialylation and desialylation status of the intracellular PBMCs protein was analyzed through Western blot followed by incubation with SNA and DSA lectins, respectively. (**A**) Total intracellular sialylation levels. (**B**) Total intracellular desialylation levels. Data represent the mean ± SEM and are expressed as relative amounts compared with healthy controls. Each dot and triangles in the graphs represent a single healthy control and a single BrS patient, respectively. Statistical significance was determined by Student’s *t*-test or Mann-Whitney, based on the normal distribution of data. CTR: N = 16, BrS: N = 16. * *p* < 0.05.

WB analysis with SNA and DSA lectins was then performed to evaluate the sialylation status of the intracellular proteins of PBMCs. Measurement of total sialylated proteins detected with SNA lectin revealed a significant decrease in BrS patients compared with the healthy controls (Figure 3A). In particular, the enhanced densitometric analysis showed a marked 2-fold reduction in both discrete protein bands at 75 and 40 kDa, respectively (Supplementary Figure S1A). Analysis of total desialylated proteins with DSA showed a significant decrease in BrS compared with CTRs (Figure 3B). Enhanced densitometric analysis showed a significant decrease in discrete protein bands at 160, 140, 80, 55, and 45 kDa by 1.7-, 2.1-, 1.7-, 3.4-, and 2.3-fold, respectively, in BrS patients compared with CTRs (Supplementary Figure S1B).

### 2.5. Analysis of Circulating Plasma Proteins Show Changes in Protein Sialylation Levels in BrS Patients

**Figure 4:**
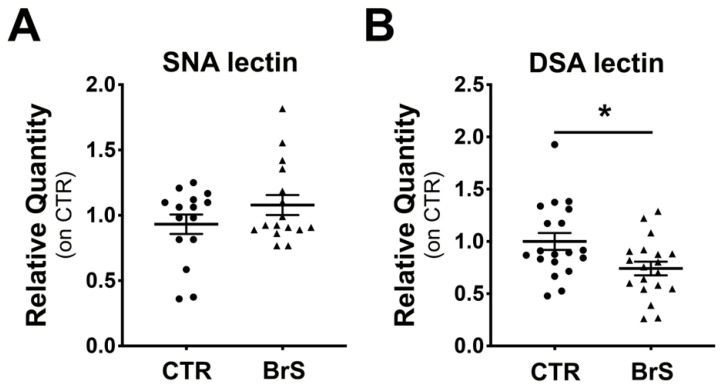
Plasma proteins showed a reduction in desialylation in BrS patients. Sialylation and desialylation status of the plasma proteins was analyzed through Western Blot followed by incubation with SNA and DSA lectins, respectively. (**A**) Total plasma sialylation levels. (**B**) Total plasma desialylation levels. CTR: N = 19, BrS: N = 19. Data represent the median ± SEM and are expressed as relative amounts compared with healthy controls. Each dot and triangles in the graphs represent a single healthy control and a single BrS patient, respectively. Statistical significance was determined by Student’s *t*-test. * *p* < 0.05.

We then investigated whether protein sialylation changes could be detected in the circulating proteins of BrS patients. For this purpose, Western blot analysis with SNA and DSA lectins was performed on plasma proteins from BrS patients and compared with healthy controls. Densitometric analysis did not show any differences in sialylated protein (Figure 4A), while a significant decrease of desialylated proteins was observed in BrS patients compared with healthy controls (Figure 4B). However, the enhanced densitometric analysis revealed two distinct bands at 65 and 55 kDa, showing a 2- and 1.8-fold reduction in sialylation in BrS compared with CTRs, respectively (Supplementary Figure S2A). In addition, three different protein bands at 125, 75, and 65 kDa showed a 1.6-, 1.4-, and 2-fold decrease in sialylation in BrS patients compared with CTR, respectively (Supplementary Figure S2B).

### 2.6. Sialyltransferases Expression Levels Correlate with the BrS Severity

**Figure 5:**
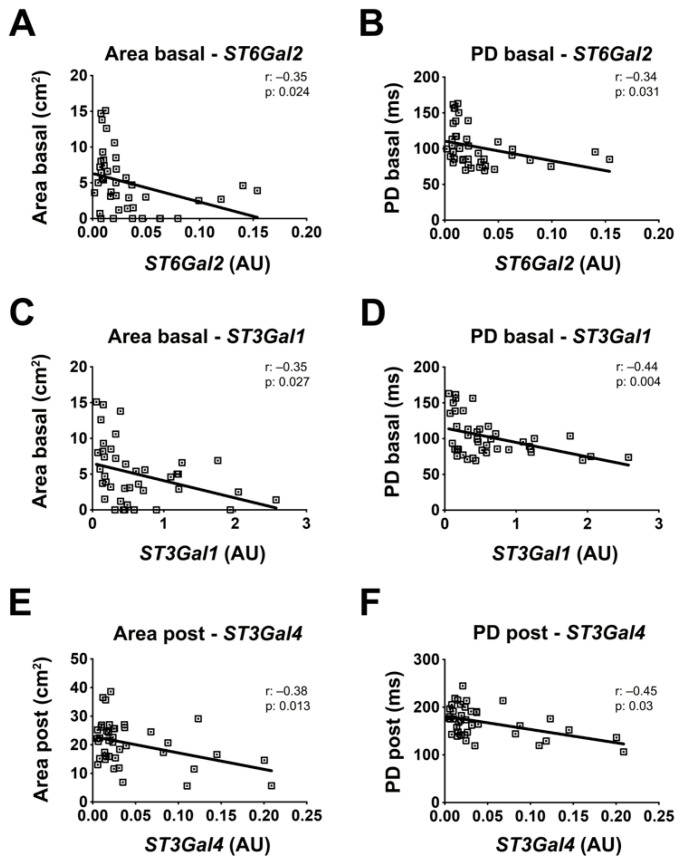
Sialyltransferases mRNA levels are correlated with BrS clinical features. The correlation between sialyltransferases gene expression and clinical features was analyzed by comparing the RNA expression levels of *ST6Gal2*, *ST3Gal1*, and *ST3Gal4* with different clinical characteristics of BrS patients. Correlation between *ST6Gal2* mRNA levels and basal pathological substrate area (**A**) and PD (**B**). Correlation between *ST3Gal2* mRNA levels and basal pathological substrate area (**C**) and PD (**D**). Correlation between *ST3Gal4* mRNA levels and post-ajmaline pathological substrate area (**E**) and PD (**F**). BrS: N = 41. Each square in the graphs represent a single BrS patient. The statistical significance of the correlation was evaluated using the Pearson’s r coefficient and the *p* value.

Finally, we performed a correlation analysis between sialyltransferases gene expression levels and several clinical characteristics of BrS patients, including sex, age, familiarity for BrS and sudden death, arrhythmogenic substrate size, and potential duration (PD), basal and after ajmaline challenge. Results showed a significant association between *ST6Gal2* expression level and both basal arrhythmogenic substrate area size and PD, with Pearson coefficients of −0.35 and −0.34 and *p* values of 0.024 and 0.031, respectively (Figure 5A,B). *ST3Gal1* was also negatively associated with substrate size and PD, with Pearson coefficients of −0.35 and −0.44 and *p* values of 0.027 and 0.004, respectively (Figure 5C,D). *ST3Gal4* levels resulted inversely connected with post-ajmaline substrate area and PD, with Pearson coefficients of −0.38 and −0.45 and *p* values of 0.013 and 0.03, respectively (Figure 5E,F). Sex, age, and familiarity for BrS and sudden death showed no significant correlation with gene expression (Supplementary Figures S3–S5).

### 2.7. Sialylation of PBMC Proteins Are Associated with the BrS Phenotype Manifestation

**Figure 6:**
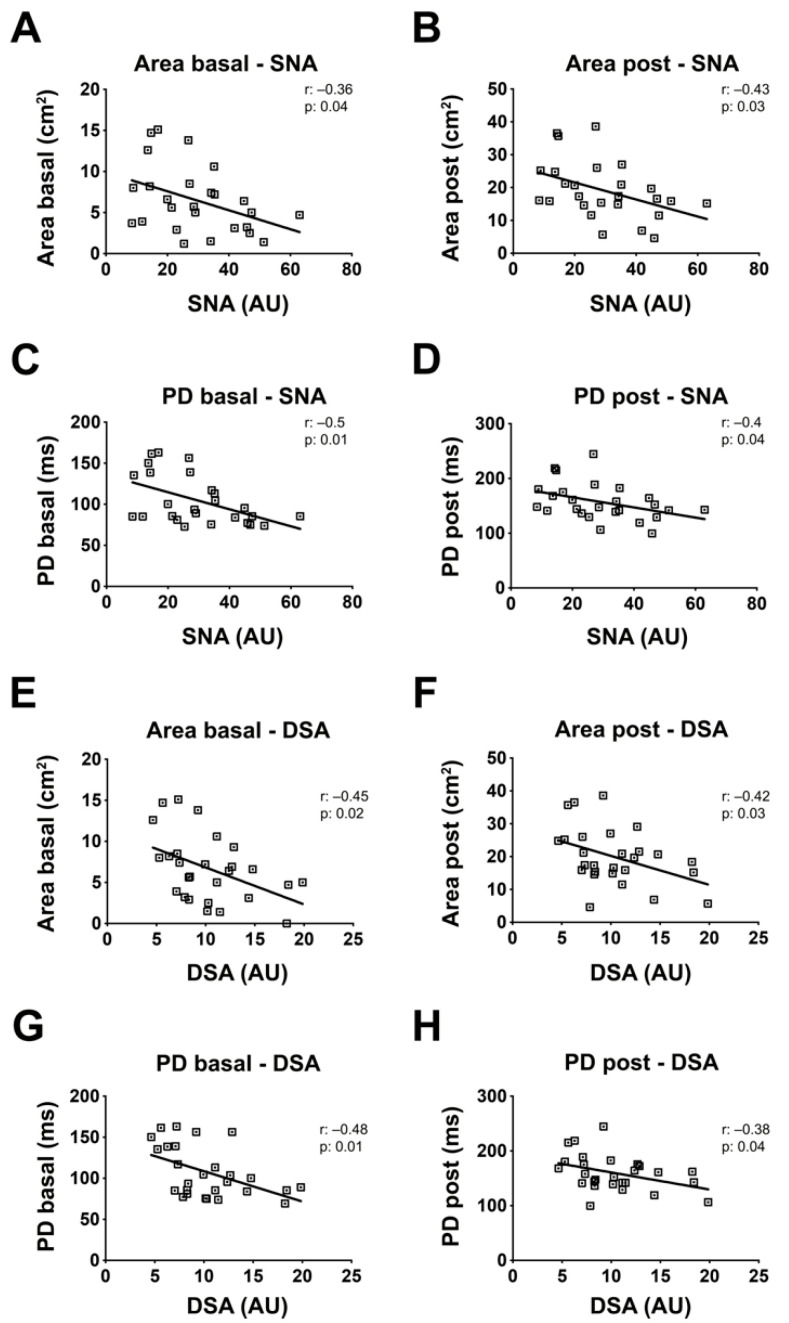
Membrane protein sialylation levels of PBMCs correlate with BrS clinical features. The correlation between sialylation with clinical features was analyzed by comparing the PBMCs proteins membrane SNA and DSA levels with different clinical parameters of BrS patients. Correlation between SNA levels and basal (**A**) and post-ajmaline (**B**) pathological substrate area, and basal (**C**) and post-ajmaline (**D**) PD. Correlation between DSA and basal (**E**) and post-ajmaline (**F**) pathological substrate area, and basal (**G**) and post-ajmaline (**H**) PD. BrS: N = 16. Each square in the graphs represent a single BrS patient. The statistical significance of the correlation was evaluated using the Pearson’s r coefficient and the *p* value.

Significant associations were found between PBMCs protein sialylation levels measured by cytofluorimetry with SNA lectin and basal and post-ajmaline arrhythmogenic substrate areas of BrS patients, with Pearson coefficients of −0.36 and −0.43 and *p* values of 0.04 and 0.03, respectively (Figure 6A,B). In addition, significant associations were found between protein sialylation levels and PD of BrS patients, with Pearson coefficients of −0.5 and −0.4 and *p*-values of 0.01 and 0.04, respectively (Figure 6C,D). Analysis of desialylated proteins of BrS patients measured by cytofluorimetry with DSA lectin revealed a significant link with both arrhythmogenic substrate area and PD with Pearson coefficients of −0.45 and −0.42 and *p*-values of 0.02 and 0.03, respectively (Figure 6E,F). The level of desialylated proteins were also connected with both basal and post-ajmaline PD, with Pearson coefficients of −0.48 and −0.38 and *p*-values of 0.01 and 0.04, respectively (Figure 6G,H). Sex, age, and familiarity for BrS and sudden death showed no significant correlation with protein sialylation (Supplementary Figures S6 and S7).

### 2.8. Inward Sodium Current Is Reduced as a Result of Desialylation

**Figure 7:**
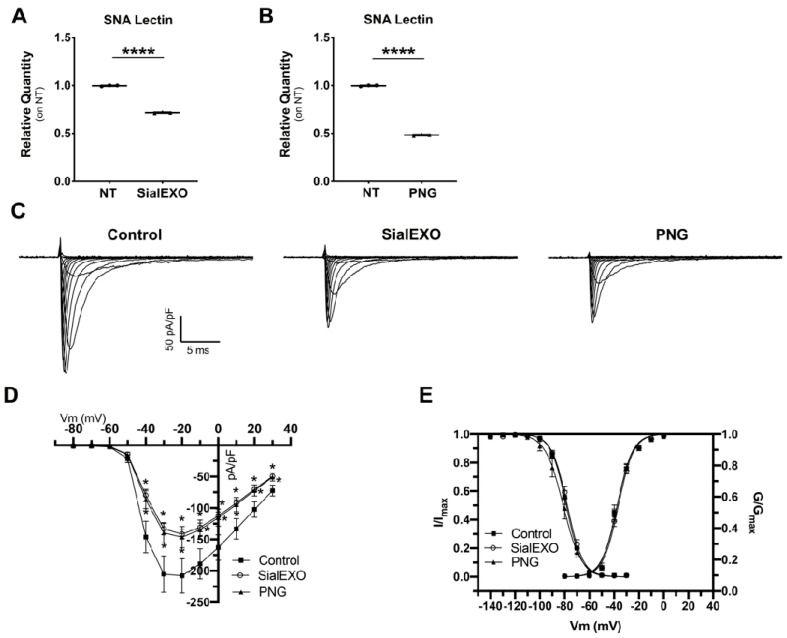
Reduced sialylation in HEK293A cell overexpressing NaV1.5 caused a decrease in inward sodium currents. (**A**) Flow cytometry analysis of membrane sialylated protein levels in HEK293A cells overexpressing NaV1.5 after SIA treatment. (**B**) Flow cytometry analysis of membrane sialylated protein levels in HEK293A cells overexpressing NaV1.5 after PNG treatment. (**C**) Representative families of current traces evoked by the protocol described in the Methods section. (**D**) Current-voltage relationship obtained for the control condition (filled square, *n* = 15), SIA and PNG incubation (empty circles, *n* = 24 and filled triangles, *n* = 14, respectively). (**E**) Voltage dependence of activation and inactivation in the three conditions tested. **** *p* < 0.0001, * *p* < 0.05 (One-way ANOVA).

To study the effects of sialic acid alterations on sodium currents, HEK293A overexpressing NaV1.5 sodium channel were treated with SialEXO and PNGase (PNG) to specifically remove sialic acid and N-glycans, respectively. Flow cytometry analysis showed a significant 1.3- (for SialEXO) and 1.5-fold (for PNG) decrease of membrane sialylated proteins (Figure 7A,B). Patch clamp experiments revealed that the incubation with SialEXO and PNG produced a significant INa density reduction (Figure 7C). In particular, INa peak density at −20 mV was 207.9 ± 27.4 pA/pF in control (*n* = 15), −141.4 ± 7.5 pA/pF pA/pF (*n* = 24) and −146.3 ± 17.0 pA/pF (*n* = 14) after 6 h in the presence of SialEXO and PNG, respectively (Figure 7D,E). No significant changes were observed in the conductance curves; the fitting of the curves with a Boltzmann function yield a voltage of half activation (V1/2) values of −37.6 ± 0.6 mV (slope 6.3 ± 0.5), −36.8 ± 0.4 mV (slope 6.2 ± 0.4) and −38.0 ± 0.5 mV (slope 6.2 ± 0.4) for control, SialEXO and PNG, respectively (Figure 7C). In a similar fashion, no significant changes were seen in the voltage dependence of steady-state inactivation. In this case, the V1/2 values were −78.6 ± 1.2 mV (slope 5.3 ± 0.1), −77.8 ± 1.1 mV (slope 5.2 ± 0.1) and −81.4 ± 2.1 mV (slope 5.3 ± 0.1) for the three conditions tested (Figure 7C).

## 3. Discussion

Recent studies have challenged the notion of Brugada Syndrome as a genetic channelopathy affecting only the human heart [26]. Indeed, genes associated with BrS, such as *SCN5A*, have been shown to be expressed throughout the body and could contribute to the occurrence of overlapping pathologies, although solid evidence of a multi-organ or systemic disease is still lacking [26]. Moreover, observations of altered plasma proteins in BrS patients were not conclusive [24,25], as they could simply reflect cardiac dysfunction [27]. In this study, we provide decisive evidence that BrS also affects extracardiac tissues, as we found significant alterations in gene expression and protein sialylation in PBMCs of affected patients. Highly sialylated proteins such as voltage-gated cardiac channels may suffer from malfunctions of this post-translational machinery. A decrease in protein sialylation has been associated with increased arrhythmic events, as proper sialylation of membrane proteins is crucial for physiological cellular excitability [23,28,29]. Consistent with these results, BrS patients showed a significant decrease in protein sialylation levels in both their plasma and PBMCs, along with altered expression of sialyltransferases. Furthermore, a direct correlation between *ST6Gal2* sialyltransferase activity and sialylated membrane proteins was observed, suggesting a causal relationship. In this regard, a role of sialylation in cardiovascular diseases has been already described. Indeed, decreased protein sialylation levels were associated with the development of dilated cardiomyopathy in the absence of a pathological stimulus [29], whereas higher levels of sialylated proteins were found in hypertrophic hearts [30]. Similarly, sialyltransferases have been found altered in ischemic myocardium [31], and overexpression of ST3Gal2 is associated with dilated cardiomyopathy [32]. Moreover, downregulation or silencing of ST3Gal4 has been correlated with a reduction in voltage-gated sodium channel function [19,33]. While more than twenty genes have been associated with BrS, the *SCN5A* is generally accepted as the causative gene of the syndrome [34]. It has also been demonstrated that *SCN5A* mutations determine a more aggressive clinical manifestation due to the larger extent of epicardial arrhythmogenic substrate compared with patients without *SCN5A* mutation, supporting its prognostic role [10]. Unfortunately, variants in the *SCN5A* gene are found in approximately 20% of all BrS probands [8], and cardiac arrest and life-threatening ventricular arrhythmias still occur in genotype-negative BrS patients [10]. Recent evidence suggests that the manifestation of BrS is due to the presence of both altered cardiac ion channel activity and structural abnormalities of the RVOT. In particular, decreased conduction reserve, in combination with the presence of abnormal interstitial fibrosis, contributes to current-load mismatch and subsequently arrhythmias [13,14]. This concept supports the hypothesis that BrS should be considered an oligo- or polygenic disorder and that multiple variants might influence the phenotype by affecting sodium channel function through different pathways [35]. Indeed, NaV1.5 activity is regulated by protein interactors and sialylation could have a role. For example, β-subunit 2 is involved in the efficient transport of NaV1.5 to the membrane and has three glycosylation sites, one of which is sialylated. If it lacks sialic acid, it will not transport NaV1.5 to the membrane [36]. β-subunit-1 also exhibits sialylation sites that affect NaV1.5 gating [37], further supporting a role for sialylation in NaV1.5 activity. Remarkably, these changes could be correlated with the phenotypic expression of the disease, namely, the extent and electrophysiological abnormalities of the epicardial arrhythmogenic substrate, which is considered the BrS phenotype [10]. The latter suggests the involvement of sialylation in the etiology of the disease and possibly its prognostic value. Because there is no reliable animal model for the syndrome [38] and we did not have access to cardiac biopsies, which would be too risky of a procedure for the patient, we tested this hypothesis by engineered HEK cells, which do not normally express NaV1.5, with a plasmid carrying a wild-type *SCN5A* construct. Removal of membrane sialic acid by treatment of cells with the enzymes glycosidase or neuraminidase resulted in a significant decrease in sodium current density, demonstrating that changes in the cell membrane sialylation strongly affect channel activity. In addition, we observed overlapping current reductions when we removed all glycans or only sialic acids, suggesting that sialylation status plays a key role in channel function. Furthermore, as mentioned before, NaV1.5 physiology is characterized by several sialylated interactors that regulate membrane localization, membrane stabilization, and activity, which probably contribute to the observed effects due to desialylation [39].

Altogether, these results provide a new pathophysiological hypothesis of sodium channel dysfunction due to PTM in BrS patients. Furthermore, because changes in sialylation were observed in circulating cells, these results suggest that Brugada Syndrome may affect tissues and organs other than the heart. This is consistent with the observation that BrS patients often have overlap with noncardiac pathologies, including epilepsy, thyroid dysfunction, cancer, diabetes, skeletal muscle channelopathies, and laminopathies [26]. Moreover, the discovery that gene expression and protein alterations can be found in the peripheral blood of BrS patients suggests the existence of a peripheral biomarker signature of the disease. It remains a grand challenge to develop an effective diagnostic test to screen broadly for the disease. In this direction, a large multiomic study is currently ongoing in our research center.

### Study Limitations

This study was conducted in a population with various clinical characteristics who were evaluated at and/or referred to an experienced BrS center because they had an increased arrhythmic risk profile. However, because nearly half of the patients had not received appropriate ICD therapy at the time of this study, we acknowledge that this population may have heterogeneous clinical characteristics. Therefore, these results may not be generalizable to other patient populations. However, this study has demonstrated for the first time the correlation between peripheral sialylation abnormalities and BrS phenotype in a cohort of *SCN5A* mutation-negative high-risk patients. The presence of sialylation abnormalities and their role in BrS patients with pathogenic *SCN5A* mutations is still unknown. Moreover, the role of non-*SCN5A* mutations was not evaluated, since it was out of the scope of this study. In addition, recent evidence suggests that BrS is an oligo/polygenic disease in which more than one molecular mechanism, thus including sialylation, may cause the manifestation of pathology. All these aspects will be the subject of future research. To assess whether the arrhythmogenic cardiac substrate of BrS patients has alterations in the sialylation machinery and if the NaV1.5 sialylation decrease is proportional to the substrate area, a biopsy of the fine layer of the outflow tract would have had to be performed, which is a risky procedure. Nevertheless, in silico analysis of the deposited transcriptomic profile (GSE93530) of cardiomyocytes derived from induced pluripotent stem cells (iPSc) from BrS patients [40] revealed alterations in several key enzymes involved in sialylation, including *ST3Gal1*, *ST3Gal4*, *ST6Gal2*, *ST8Sia4*, and *ST8Sia5* (Supplementary Figure S8). However, sialylation is known to change drastically during embryogenesis, especially in the heart [41]. Therefore, cardiomyocytes from iPSCs may also not represent a suitable model to study the sialylation status of an adult human heart due to the current limitations in their maturation status [42,43,44]. In addition, previous studies have shown that cardiomyocytes from iPSCs of patients without *SCN5A* mutations did not have significant differences in sodium currents compared with controls [45,46], further emphasizing the need to develop more complex in vitro and in vivo models to recapitulate and study this pathology.

## 4. Materials and Methods

### 4.1. Study Population

Subjects referred to the Arrhythmology Department of IRCCS Policlinico San Donato for suspected BrS were enrolled in this study. Physical examination, baseline ECG, and medical history were collected for all individuals. Patients were classified using the Shanghai Score System [47]. The subject not presenting a spontaneous BrS type 1 ECG underwent an ajmaline challenge. The study population was composed of two separate groups: the Brugada group (BrS) consisted of patients affected by BrS according to the most recent Expert Consensus Conference [48]. Since they were deemed as high-risk patients, with a negative genetic test for *SCN5A* variants and the BrS panel (*SCN10A*, *SCN1B*, *SCN2B*, *SCN3B*, *RANGRF*, *GPD1L*, *CACNA1C*, *CACNA2D1*, *CACNB2*, *TRPM4*, *PKP2*, *ABCC9*, *HCN4*, *KCND2*, *KCND3*, *KCNE3*, *KCNE5*, *KCNJ8*, *TPM1*, *MYBPC3*, *SEMA3A*, *FGF12*, *SLMAP*, *HEY2*, *LRRC10*) [12], a phenotype evaluation by epicardial mapping was performed. The control group (CTR) was defined as individuals who tested negative for BrS by a sodium channel blocker (SCB) test with ajmaline. A drug challenge was performed according to consensus criteria [48]. All subjects were not under active pharmacological treatment.

### 4.2. Electrophysiological Study and Arrhythmogenic Substrate Mapping

An electrophysiological study (EPS) was systematically performed as previously described, only in BrS patients [10]. All patients underwent a combined endo-epicardial mapping procedure using a three-dimensional (3D) mapping system (CARTO 3, Biosense Webster, CA, USA). Epicardial access was obtained using fluoroscopy-guided subxyphoidal puncture, and a steerable sheath (Agilis EPI, Abbott, MN, USA) was introduced. All maps were obtained at baseline conditions (Supplementary Figure S9) and after ajmaline test (up to 1 mg/kg over 10 min) (Supplementary Figure S10). Ajmaline was administered (1) to achieve the maximal ST-segment elevation and unmask, if necessary, the type 1 ECG pattern and (2) to identify the real extent of the regions displaying fragmented and abnormal electrograms (EGMs). The potential duration maps (PDM) were performed by collecting the duration of each bipolar EGM filtered from 16 Hz to 500 Hz, displayed at 200 mm/s speed, and collected between the distal electrode pair. The abnormal epicardial EGMs were identified if they met at least one of the following characteristics: (i) a wide duration (>110 ms) with fragmented component (>3 distinct peaks); (ii) late component of low-voltage amplitude ranging from 0.05 to 1.5 mV; (iii) distinct and delayed component exceeding the end of the QRS complex; and (iv) discrete double activity. Total signal duration was measured for each potential before and after drug challenge as previously described (10). The potential duration map was created by collecting the duration of each EGM. As a result, a color-coded map was obtained showing the regions displaying the shortest (red color) and the longest (purple color) durations. Arrhythmogenic substrate areas were measured and validated by two expert electrophysiologists using CARTO3 system, both blinded to the sialylation analysis.

### 4.3. Plasma Isolation and Processing

Plasma was isolated from 5 mL of peripheral venous blood by centrifugation at 1500 rpm for 15 min. The collected plasma was then processed to remove the most abundant proteins using the Multiple Affinity Removal Spin Cartridge (Agilent Technologies, Santa Clara, CA, USA), following the manufacturer’s instructions. Total protein levels were determined using the Pierce BCA assay (Thermo Scientific, Waltham, MA, USA) according to the manufacturer’s instructions. All samples were collected under the same conditions and processed within 30 min to avoid or minimize any bias.

### 4.4. Isolation and Processing of Peripheral Blood Mononuclear Cells

PBMCs were isolated from 5 mL peripheral venous blood using Histopaque^®^-1077 (GE-Healthcare, Chicago, IL, USA) according to the manufacturer’s instructions. After isolation, PBMCs were washed twice in phosphate buffer saline (PBS) and centrifuged at 400× *g* for 10 min at room temperature. Cell viability was determined using the trypan blue exclusion assay. Aliquots of PBMCs were collected from each subject and processed for protein and/or RNA extraction and/or flow cytometry analysis. All samples were collected under the same conditions and processed within 30 min to avoid or minimize any bias.

### 4.5. Flow Cytometry Analysis

About 1 × 10^6^ of PBMCs were stained with two different FITC-conjugated lectins: SNA, Sambucus Nigra Lectin from elderberry bark (EY Laboratories, San Mateo, CA, USA) that preferentially recognizes sialic acid linked (α-2,6) to galactose and to a lesser degree the (α-2,3) linkage, and DSA, Datura Stramonium Lectin which recognizes galactose linked (β-1,4) to N-acetyl-glucosammine. Briefly lectins were diluted in the indicated buffer (0.01 M Phosphate, 0.15 M NaCl, pH 7.2–7.4), 100 μg/mL. Cells were washed in the same buffer, centrifuged, and the pellet was resuspended in 1 mL of diluted lectin solution and incubated 15 min at Room Temperature (RT). Finally, cells were washed three times with the buffer, by centrifugation, and analyzed in 300 μL of buffer. Unstained cells were used to assess a baseline. Sample acquisition was performed using a Navios flow cytometer (Beckman Coulter, Brea, CA, USA) and analyzed using Kaluza software (Beckman Coulter, Brea, CA, USA).

### 4.6. Real-Time PCR

RNA was extracted from PBMCs with ReliaPrep™ RNA Miniprep System (Promega, Madison, WI, USA), following the manufacturer’s instructions. Then, 1 μg of RNA was reverse transcribed to cDNA with the iScript cDNA synthesis kit (Bio-Rad, Hercules, CA, USA), according to the manufacturer’s instructions. Real-time PCR was performed with 10 ng of cDNA template, 0.2 μm primers, and 1 × GoTaq^®^ qPCR Master Mix (Promega, Madison, WI, USA) in 20 μL of final volume, using a StepOnePlus^®^ real-time PCR system (Applied Biosystem, Waltham, MA, USA). The amplification protocol was: 95 °C for 2 min, 40 cycles of 5 s each at 95 °C, 30 s at 57 °C and 30 s at 72 °C, and a final stage at 72 °C for 2 min. Relative quantification of target genes was calculated by the equation 2^−ΔΔCt^ using HPRT as the housekeeper gene. The primer sequences used were:

- *ST3Gal1* FW: 5′-CGGGAGCTGGGAGATAATGT-3′
- *ST3Gal1* Rev: 5′-TGATGAAGGCTGGGTGGTAG-3′
- *ST3Gal4* FW: 5′-TCCTGGTAGCTTTCAAGGCA-3′
- *ST3Gal4* Rev: 5′-CAGGCTCAGCAGTTTGTC-3′
- *ST6Gal2* FW: 5′-CCCCAGCCATCACTTCATTG-3′
- *ST6Gal2* Rev: 5′-TGGGTTTCTCTGACGATGCT-3′
- *Neu3* FW: 5′-TGGTCATCCCTGCGTATACC-3′
- *Neu3* Rev: 5′-TCACCTCTGCCACTTCACAT-3′
- *CMAS* FW: 5′-CTGTTGTGAGACGCCATCAG-3′
- *CMAS* Rev: 5′-CCACACTATGTTCAGCTCGC-3′
- *GNE* FW: 5′-GTGGTACTTGGCTCTCACCT-3′
- *GNE* Rev: 5′-ATCTGGCAGCTTCACTAGGG-3′
- *SLC17A5* FW: 5′-TGGAGGATATGTTGCCAGCA-3′
- *SLC17A5* Rev: 5′-GAGCCCAAGAAGACCACATG-3ì
- *HPRT* FW: 5′-TATGGCGACCCGCAGCCCT-3′
- *HPRT* Rev: 5′-CATCTCGAGCAAGACGTTCAG-3′

### 4.7. Western Blot

For protein extraction, PBMCs were lysed with RIPA buffer (1% Nonidet P-40 in 50 mm Tris-HCl, pH 7.5, 150 mm NaCl, 0.1% sodium deoxycholate, 1% protease inhibitor cocktails), incubated in ice for 30 min, and then centrifuged at 13,000× *g* for 15 min at 4 °C. The supernatant was collected, and the total amount of proteins was determined with Pierce BCA assay (Thermo Scientific, Waltham, MA, USA), following the manufacturer’s instructions. Then, PBMCs or plasma proteins (20 µg) were resolved on a 10% SDS-PAGE gel and subsequently transferred onto nitrocellulose membranes by electroblotting. The total amount of transferred proteins used for the normalization was determined with the REVERT Total Protein Stain kit (LI-COR Biotechnology, Lincoln, NE, USA), following the manufacturer’s instructions. For the detection of sialylated or desialylated proteins, the DIG Glycan Differentiation kit (Roche, Basel, Switzerland) was employed. Briefly, membranes were incubated in Blocking solution^®^ for 30 min at RT. After two washes of 10 min in Tris Buffered Saline (TBS) (0.05 M Tris-HCl, 0.15 M NaCl, pH 7.5) and one in Buffer 1 (1 mM MgCl2, 1 mM MgCl2, 1 mM CaCl2, pH 7.5 in TBS), membranes were incubated with the specific lectins (1 μg/mL in Buffer 1) for 1 h at RT. SNA and DSA lectins were used for sialylated and desialylated proteins, respectively. Then, after three washes of 10 min in TBS, membranes were incubated with anti-Digoxigenin-AP (0.75 U/mL in TBS) for 1 h at RT. After three additional 10-min washes in TBS, membranes were stained by immersion for 5 min in the staining solution (0.1 M Tris-HCl, 0.05 M MgCl2, 0.1 M NaCl, pH 9.5, 1:50 NBT/BCIP solution). Membranes were rinsed 5 times in double distilled water and then acquired with a scanner (Canon, Ota, Tokyo, Japan). Optical density was measured with Image Studio™ Lite software (LI-COR Biotechnology, Lincoln, NE, USA).

### 4.8. Cell Culture and Transfection

HEK293 (human embryonic kidney 293) cells were cultured in a controlled environment (5% CO_2_, 37 °C) and maintained in an appropriate medium (DMEM/F12) (Euroclone, Milan, Italy) supplemented with 10% FBS, 2 mM L-Glutammine, 100 U/mL, and 100 μg/mL Pen/Strep. Wild-Type *SCN5A* complementary DNA (cDNA) was subcloned into the pcDNA3.1 plasmid, as previously described [49]. *SCN5A* was transiently transfected using Viafect reagent (Promega, Madison, WI, USA) according to the manufacturer’s instructions.

### 4.9. Sialic Acid Removal

Sialic acid was enzymatically removed from NaV1.5 overexpressing HEK293A cells with PNGase F (PNG) (Thermo Scientific, Waltham, MA, USA) or SialEXO (Genovis, Lund, Sweden). PNGase F released all the N-glycans, while SialEXO specifically removed only the sialic acid. Cells were plated at 50–60% confluency. After 24 h, cells were treated for 6 h with PNG or SialEXO diluted in cell medium at a final concentration of 0.5 units/μL and 0.8 units/μL, respectively.

### 4.10. Electrophysiological Measurements

Automated planar patch-clamp experiments were conducted with the Patchliner system (Nanion Technologies GmbH, Munchen, Germany) on NaV1.5 overexpressing HEK293 cell lines. Whole-cell recordings of INa were performed at RT with medium resistance NPC-16 chips. In order to limit the amplitude of overexpressing currents, all experiments were performed with NMDG-based low-sodium (80 mM) extracellular solutions and CsF-based intracellular solution (Nanion Technologies GmbH, Munchen, Germany). Just before the current measurements, treated cells were tripsinized, resuspended in the extracellular solution and incubated at 4 °C for 20 min to improve the membrane stability before feeding them to the Patchliner. NaV1.5 currents were elicited by a ladder voltage protocol composed by 15 incremental 10 mV steps of 50 ms duration spanning between −80 and +60 mV from a holding potential of −120 mV. From this protocol, the current-voltage relationships and the conductance curves were derived. In order to study the voltage dependence of steady-state inactivation, the cells membrane was stepped between −140 and +10 mV (10 mV increment, 500 ms duration) before a −10 mV test pulse of 20 ms duration. All tests and incubations were performed on three independent experimental days. Raw traces recorded by HEKA amplifiers with the PatchMaster software (HEKA Elektronik, Lambrecht, Germany) were exported with a home-developed tool and analyzed using Clampfit 10.7 (Molecular Devices, San Jose, CA, USA) and Origin Pro (OriginLab, Northampton, MA, USA). Both conductance and inactivation curves were fitted with a Boltzmann equation.

### 4.11. Statistical Analysis

Data are presented as mean ± SEM. Shapiro-Wilk test was used to check the normal distribution of data. The Student’s *t*-test or the one-way ANOVA was used to determine significance for normally distributed data, while the Mann-Whitney test was used for data with no Gaussian distribution. *p* values of less than 0.05 were considered to be significant. The correlation analysis was evaluated by Pearson test.

### 4.12. Study Approval

The study protocol was reviewed and approved by the local Institutional Ethic Committee (protocol BASED), and all participants provided written informed consent to participate. The study conformed to the principles of the Helsinki Declaration. All authors had full access to all data in the study and take responsibility for its integrity and data analysis.

## Figures and Tables

**Table 1:** Clinical characteristics of the study population.

	Study Sample (*n* = 84)	Patient Group (*n* = 42)	Control Group (*n* = 42)	*p*-Value ^§^
**Male, *n* (%)**	62 (73.8)	35 (83.3)	27 (64.3)	0.287
**Age (years) (mean +/− SD)**	40.2 ± 15.5	38.6 ± 12.4	40.5 ± 15.3	0.193
**Spontaneous type 1 pattern, *n* (%)**	19 (22.6)	19 (45.2)	0	<0.001
**Fever induced type 1 pattern, *n* (%)**	5 (6.0)	5 (12.0)	0	0.113
**SCA, *n* (%)**	9 (10.7)	9 (21.4)	0	<0.001
**Spontaneous VT/VF requiring ICD therapy, *n* (%)**	18 (21.4)	18 (42.8)	0	<0.001
**Nocturnal agonal respirations, *n* (%)**	4 (4.7)	4 (9.4)	0	0.113
**Suspected arrhythmic syncope, *n* (%)**	10 (11.9)	7 (16.6)	3 (7.1)	0.708
**Unclear syncope, *n* (%)**	16 (19.0)	9 (21.4)	7(16.6)	0.556
**AF/Flutter, *n* (%)**	16 (19.0)	11 (26.2)	5 (16.1)	0.365
**Family history of BrS, *n* (%)**	30 (35.7)	20 (47.6)	10 (23.8)	<0.001
**Family history of SD, *n* (%)**	37 (44.0)	23 (54.7)	10 (23.8)	<0.001
**Shanghai Score, *n* (%)**				
**Score < 3**	35 (41.6)	0	35 (83.2)	<0.001
**Score < 4**	7 (8.3)	5 (11.2)	2 (4.7)	1.000
**Score < 5**	8 (9.5)	4 (9.5)	4 (9.5)	0.053
**Score < 6**	5 (5.9)	4 (9.5)	1 (2.3)	1.000
**Score < 7**	6 (7.1)	6 (14.2)	0	0.001
**Score ≥ 7**	23 (27.4)	23 (54.8)	0	<0.001

^§^ Student’s *t*-test (two-tailed) or χ^2^ test comparing data distribution between the BrS patient and control groups.

## Data Availability

The raw data supporting the conclusions of this manuscript will be made available by the authors, without undue reservation, to any qualified researcher.

## Supplementary Materials

The following supporting information can be downloaded at: https://www.mdpi.com/article/10.3390/ijms232113154/s1.
