# Supplementary material for: Alterations of the Sialylation Machinery in Brugada Syndrome
Source: Int J Mol Sci. 2022 Oct 29;23(21):13154. doi: 10.3390/ijms232113154 (PMC9655504; doi:10.3390/ijms232113154)
Supplement: Supplementary file 1 [file ijms-23-13154-s001.zip › ijms-1943495-supplementary.pdf]

Alterations of the sialylation machinery in Brugada Syndrome

Supplemental Figures and Legends

Suppl. Figure S1

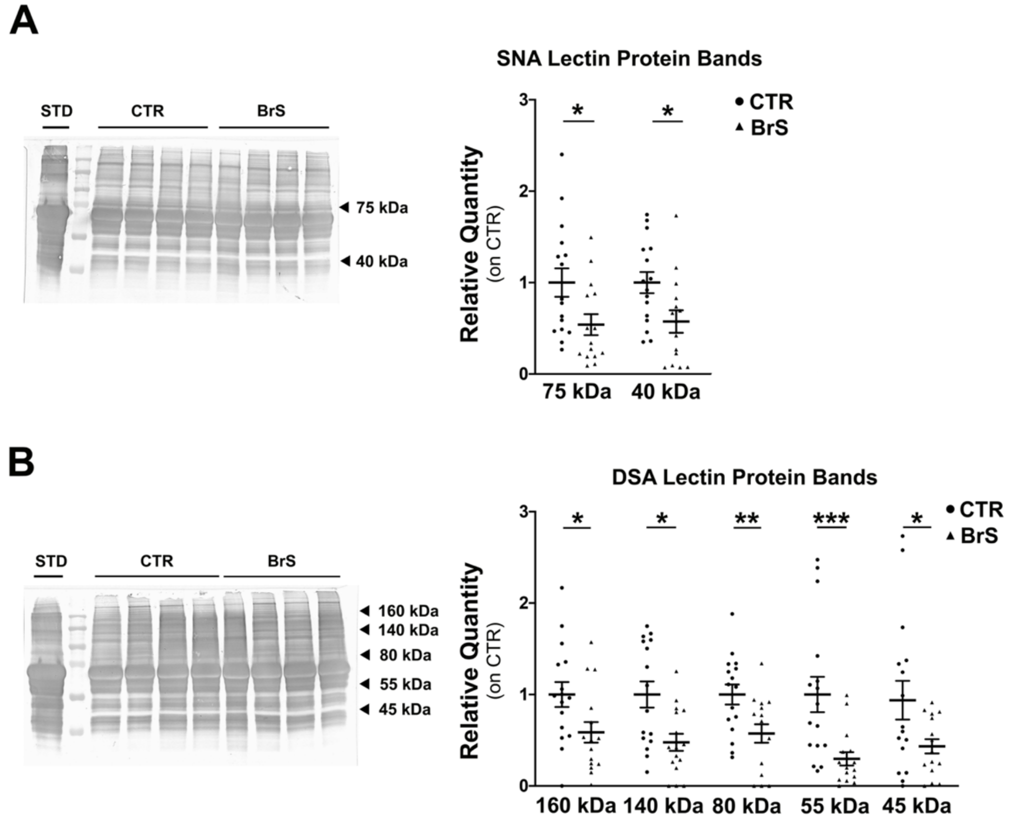

Figure S1. Molecular weight protein sialylation analysis in PBMCs reveals significant reduction of specific protein bands in BrS patients.

(A) Representative blot of PBMCs intracellular proteins after incubation with SNA lectin. Densitometric analysis of three different protein bands at 75 kDa, and 40 kDa, respectively. (B) Representative blot of PBMCs intracellular proteins after incubation with DSA lectin. Densitometric analysis of five different protein bands at 160 kDa, 140 kDa, 80 kDa, 55 kDa, and 45 kDa, respectively.

Suppl. Figure S2

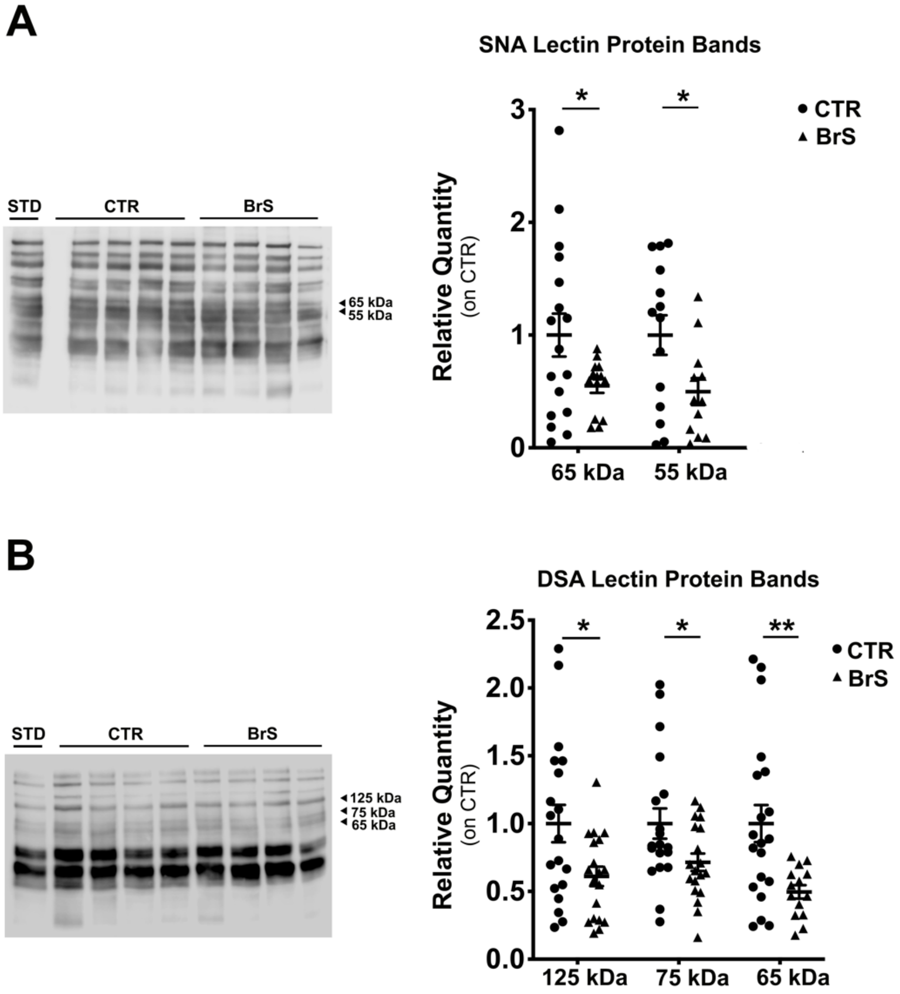

Figure S2. Molecular weight protein sialylation analysis in plasma reveals significant reduction of specific protein bands in BrS patients.

(A) Representative blot of plasma proteins after incubation with SNA lectin. Densitometric analysis of three different protein bands at 65 kDa, and 55 kDa, respectively. (B) Representative blot of PBMCs intracellular proteins after incubation with DSA lectin. Densitometric analysis of three different protein bands at 125 kDa, 75 kDa, 80 kDa, 65 kDa, and 45 kDa, respectively.

Suppl. Figure S3

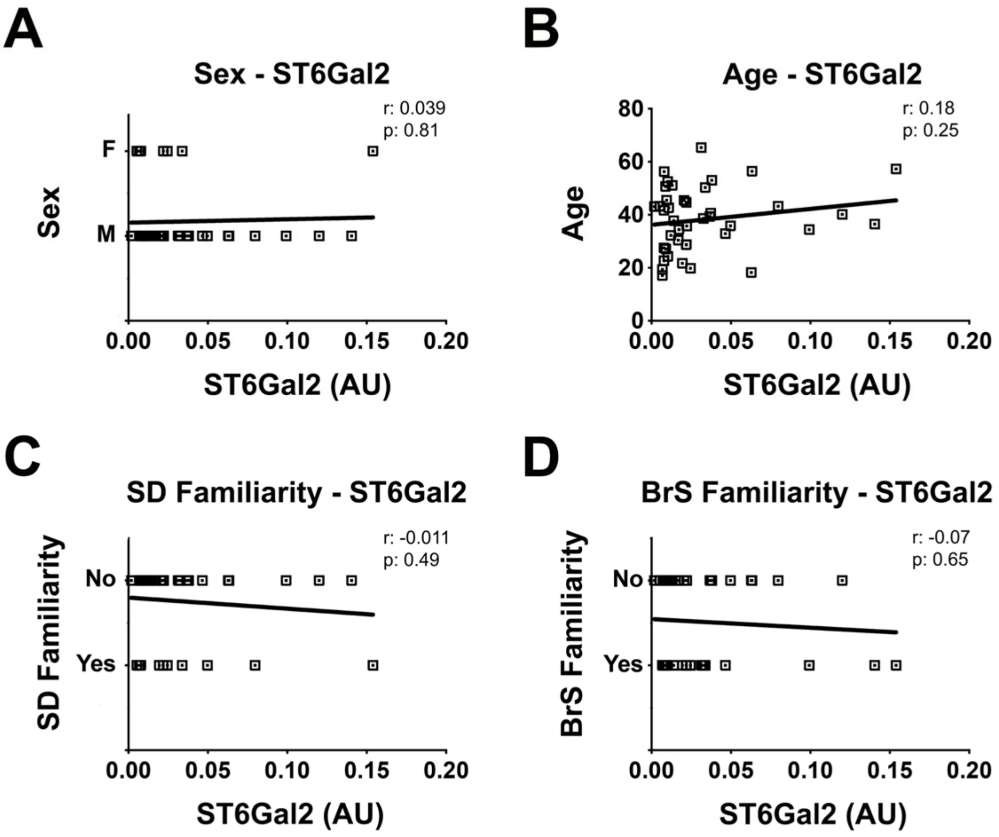

Figure S3. Correlation between ST6Gal2 mRNA expression and clinical features in BrS PBMCs.

The correlation between ST6Gal2 with clinical features was analyzed by comparing the PBMCs ST6Gal2 mRNA expression with different clinical parameters of BrS patients. Correlation between ST6Gal2 levels and sex (A), age (B), SD familiarity (C), and BrS familiarity (D) BrS: N=31. The statistical significance of the correlation was evaluated using the Pearson's r coefficient and the p value.

Suppl. Figure S4

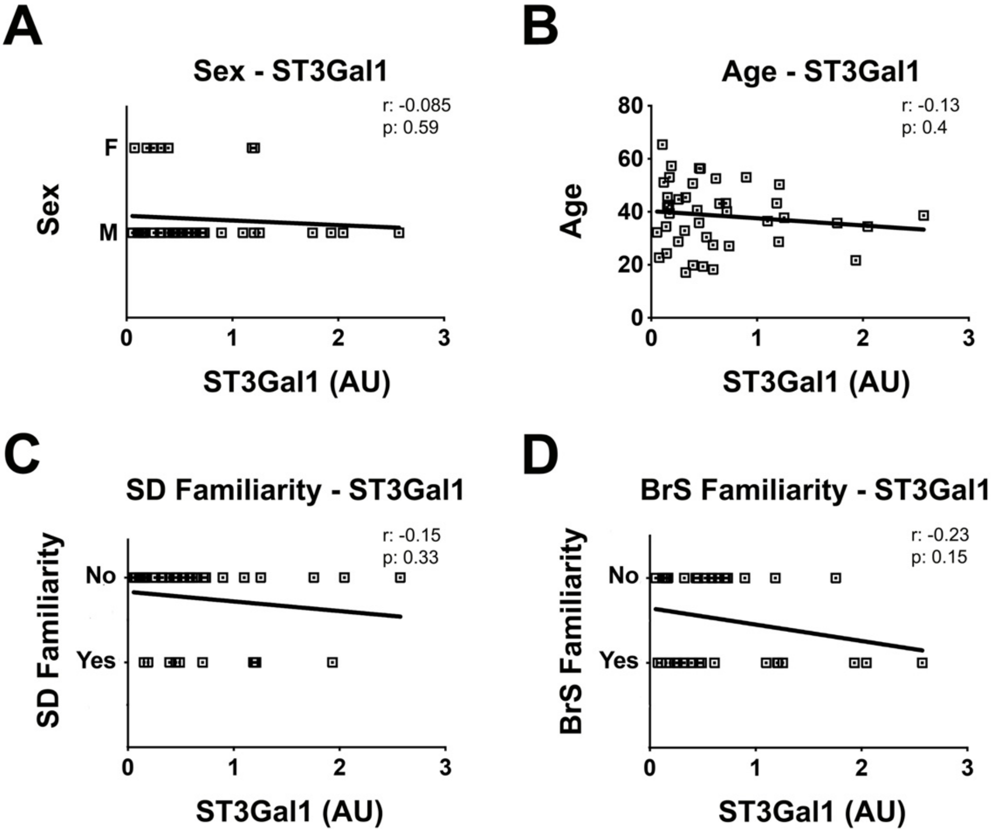

Figure S4. Correlation between ST3Gal1 mRNA expression and clinical features in BrS PBMCs.

The correlation between ST3Gal1 with clinical features was analyzed by comparing the PBMCs ST3Gal1 mRNA expression with different clinical parameters of BrS patients. Correlation between ST3Gal1 levels and sex (A), age (B), SD familiarity (C), and BrS familiarity (D) BrS: N=31. The statistical significance of the correlation was evaluated using the Pearson's r coefficient and the p value.

Suppl. Figure S5

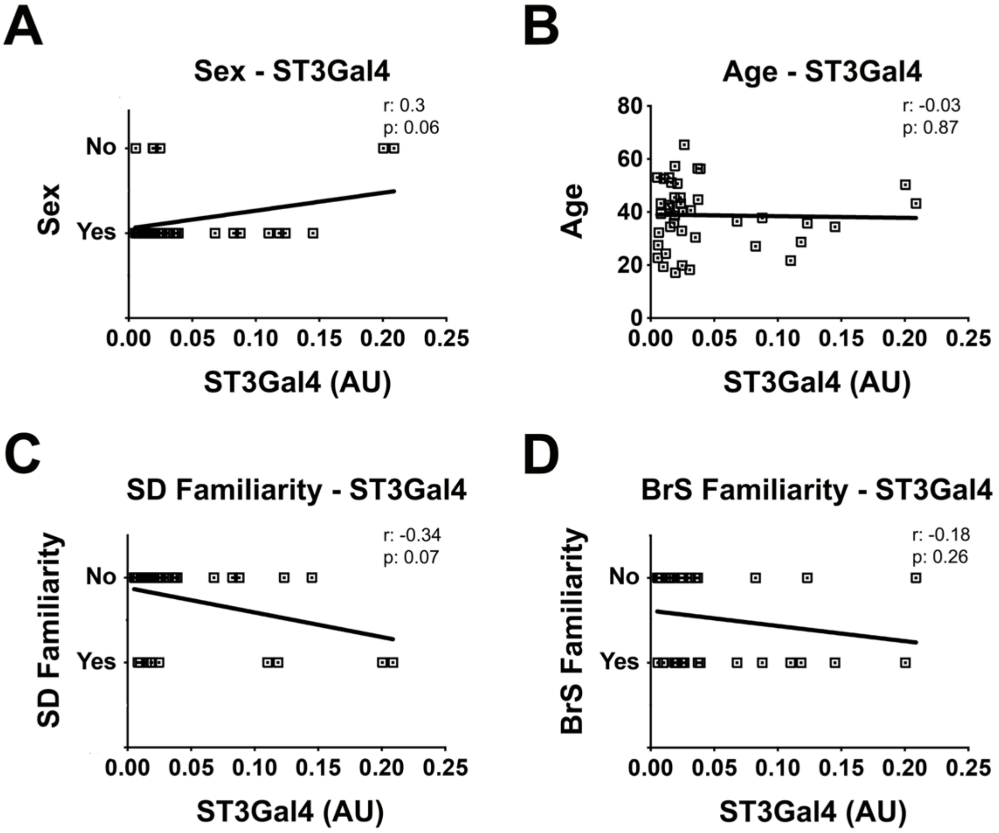

Figure S5. Correlation between ST3Gal4 mRNA expression and clinical features in BrS PBMCs. The correlation between ST3Gal4 with clinical features was analyzed by comparing the PBMCs ST3Gal4 mRNA expression with different clinical parameters of BrS patients. Correlation between ST3Gal4 levels and sex (A), age (B), SD familiarity (C), and BrS familiarity (D) BrS: N=31. The statistical significance of the correlation was evaluated using the Pearson's r coefficient and the p value.

Suppl. Figure S6

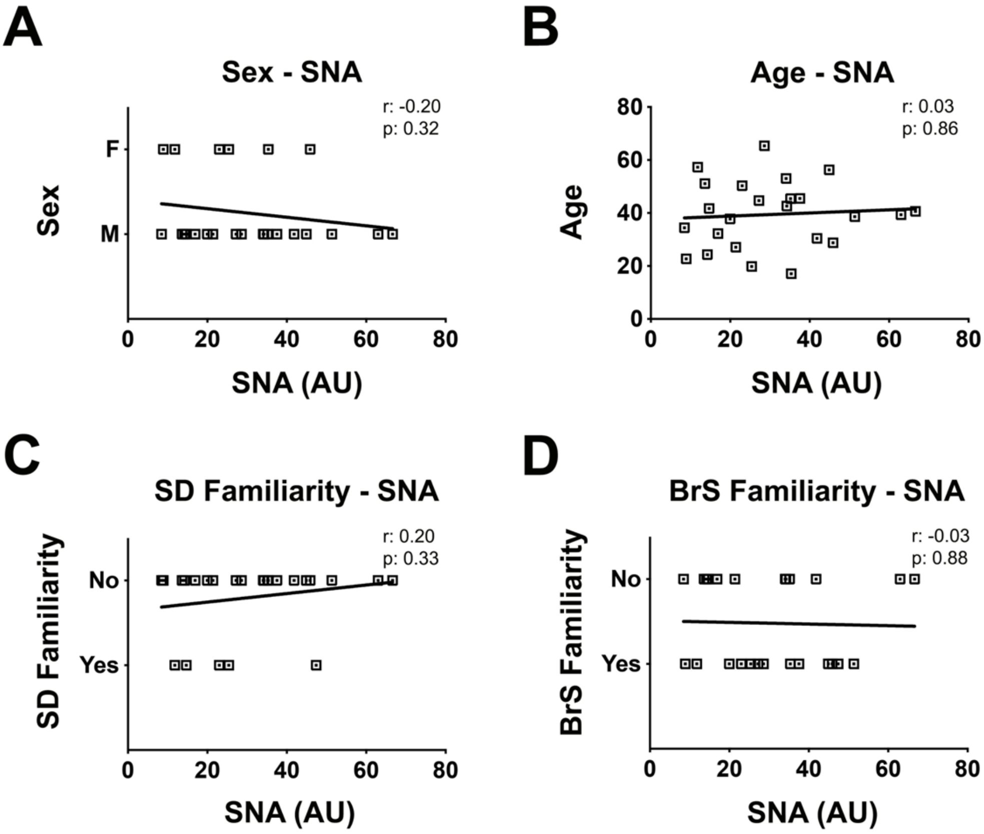

Figure S6. Correlation between protein sialylation and clinical features in BrS PBMCs.

The correlation between sialylation with clinical features was analyzed by comparing the PBMCs proteins membrane SNA levels with different clinical parameters of BrS patients. Correlation between SNA levels and sex (A), age (B), SD familiarity (C), and BrS familiarity (D) BrS: N=16. The statistical significance of the correlation was evaluated using the Pearson's r coefficient and the p value.

Suppl. Figure S7

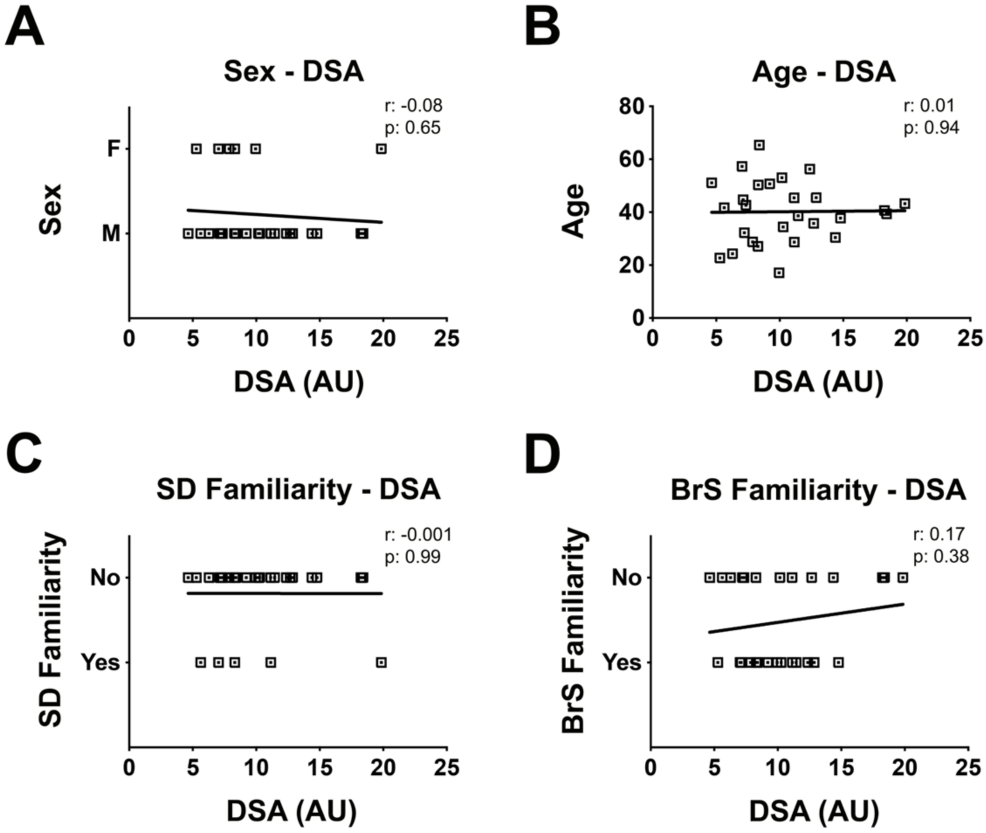

Figure S7. Correlation between protein desialylation and clinical features in BrS PBMCs.

The correlation between desialylation with clinical features was analyzed by comparing the PBMCs proteins membrane DSA levels with different clinical parameters of BrS patients. Correlation between DSA levels and sex (A), age (B), SD familiarity (C), and BrS familiarity (D) BrS: N=16. The statistical significance of the correlation was evaluated using the Pearson’s r coefficient and the p value.

Suppl. Figure S8

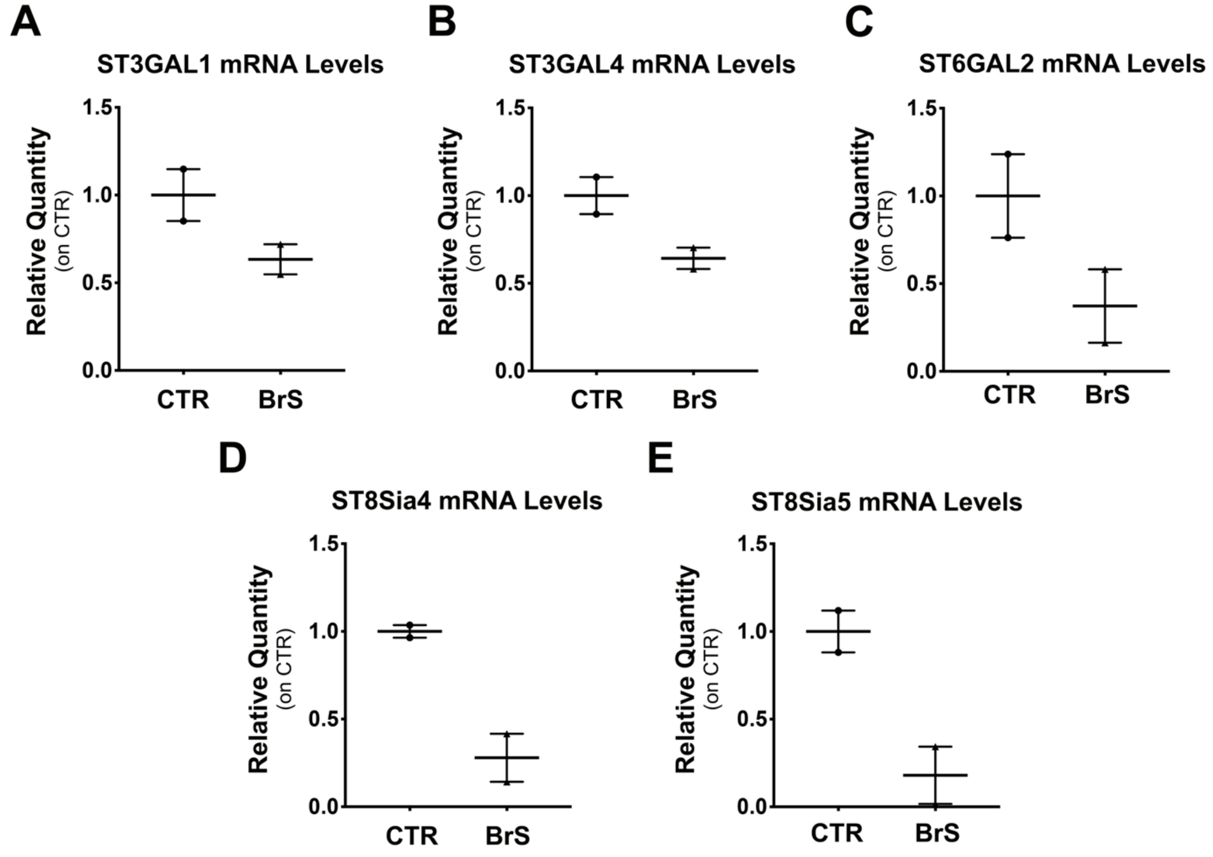

Figure S8. Gene expression analysis of sialylation pathway genes in iPS-derived cardiomyocytes. Cardiomyocytes were derived from fibroblasts of healthy subjects and BrS patients. (A) mRNA expression of ST3Gal1 through RNA-seq. (B) mRNA expression of ST3Gal4 through RNA-seq. (C) mRNA expression of ST6Gal2 through RNA-seq. (D) mRNA expression of Neu3 through RNA-seq.

(E) mRNA expression of ST8Sia4 through RNA-seq. (F) mRNA expression of ST8Sia5 through RNA-seq.

Suppl. Figure SS9

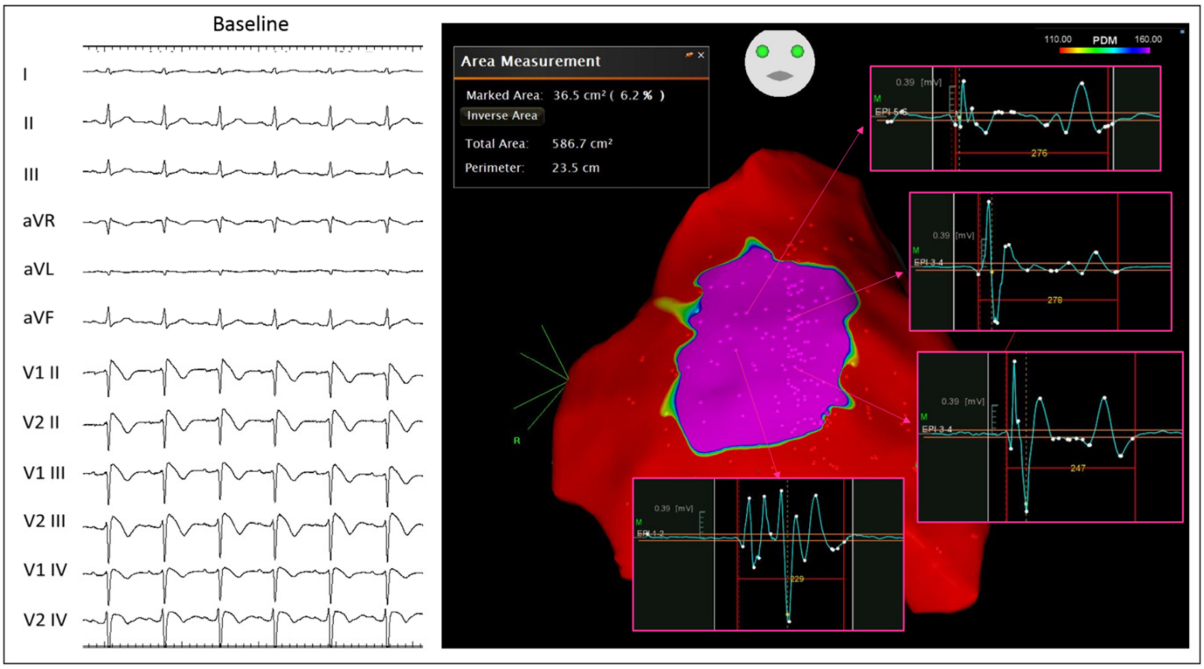

Figure S9. Epicardial mapping of spontaneous type 1 BrS pattern.

BrS male patient with spontaneous type 1 BrS pattern (ECG left panel), surviving a previous cardiac arrest and experiencing several appropriate ICD shocks. Epicardial mapping identified a large arrhythmogenic substrate (36.5 cm^2) characterized by abnormally prolonged and fragmented electrograms (CARTO map and significant abnormal electrograms on the right).

Suppl. Figure S10

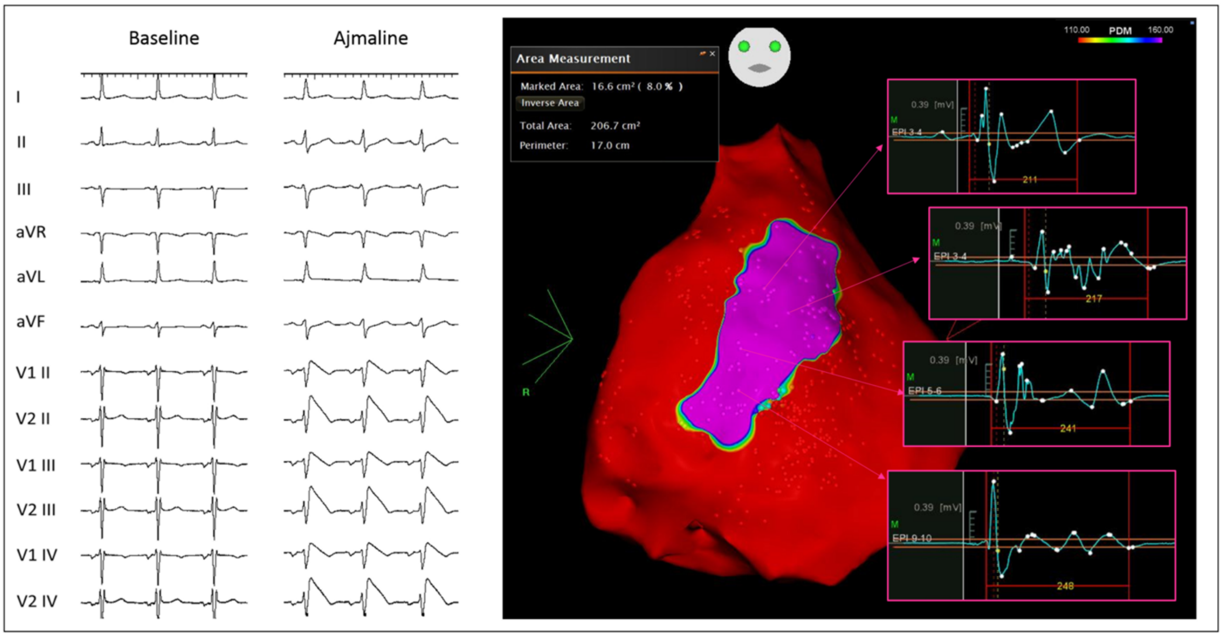

Figure S10. Epicardial mapping of drug-induced type 1 BrS pattern.

BrS male patient with drug-induced type 1 ECG pattern (left panel), with inducible VT/VF ant the EP study. He experienced spontaneous VT/VF requiring ICD shocks. Epicardial mapping demonstrated a large substrate area 16.6 cm^2 characterized by abnormal electrograms (right panel).
